# A study protocol of the rehabilitative efficacy of cardiovascular ultrasound therapy after percutaneous coronary intervention in patients with coronary artery disease: A multicenter, parallel-group, randomized controlled study

**DOI:** 10.1371/journal.pone.0327557

**Published:** 2025-10-16

**Authors:** Chunwei He, Jie Peng, Hong Liu, Lin Zhong, Yanjin Wei, Hao Qiu, Chuanliang Liu, Nana Lv, Lixia Liu, Xuewen Qi, Fenglei Zhang, Beian You, Qilong Song, Lin Shen

**Affiliations:** 1 Department of Geriatric Medicine & Laboratory of Gerontology and Anti-Aging Research, Qilu Hospital of Shandong University, Jinan, Shandong, China; 2 Qingdao Municipal Hospital, Qingdao, Shandong, China; 3 Yantai Yuhuanding Hospital, Yantai, Shandong, China; 4 Linyi People’s Hospital, Linyi, Shandong, China; 5 Tengzhou Central People’s Hospital, Tengzhou, Shandong, China; 6 Weifang People’s Hospital, Weifang, Shandong, China; 7 Jining No. 1 People’s Hospital, Jining, Shandong, China; 8 Weihai Central Hospital, Weihai, Shandong, China; 9 Liaocheng People’s Hospital, Liaocheng, Shandong, China; 10 Dongying People’s Hospital, Dongying, Shandong, China; 11 Qilu Hospital of Shandong University, Qingdao, Shandong, China; 12 Department of Emergency Medicine, Qilu Hospital of Shandong University, Jinan, Shandong, China; Al Nasiriyah Teaching Hospital, IRAQ

## Abstract

**Background:**

Coronary artery disease (CAD) is a leading cause of mortality and disability, placing heavy burdens on healthcare systems. Although cardiovascular ultrasound therapy has demonstrated effectiveness and safety in improving refractory angina, relevant clinical studies are rare and clinical evidence is lacking.

**Methods and design:**

This is a prospective, parallel-group, randomized controlled trial. We enrolled 200 patients with coronary artery disease who had undergone percutaneous coronary intervention (PCI) and randomized them into two groups. The intervention group will be given usual-practice plus cardiovascular ultrasound therapy intervention and the control group will be given only usual-practice intervention. After 20 treatments with cardiovascular ultrasound therapy, high‐sensitivity C‐reactive protein in serum will be used as the primary outcome measures. The following will be used in determining secondary outcomes: indicators of serum myocardial injury, blood lipid levels, markers of endothelial function, inflammatory factors, hemodynamic indicators, echocardiography, ultrasound examination for carotid plaques, 6-minute walk test, short-term variability in heart rate, and mental health assessment. The researchers plan to test the outcome indicators at multiple time points. Statistical analyses will be performed using SPSS version 26 statistical software (IBM, Armonk, NY).

**Discussion:**

This is the first clinical study of the rehabilitative efficacy of cardiovascular ultrasound therapy in the treatment of CAD after PCI. Clinical recovery currently depends mainly on modalities such as medication, exercise, and nutritional therapy; therefore, cardiovascular ultrasound therapy, as a new mode of therapy, might become a major advance in the treatment of CAD after PCI.

**Trial registration:**

ClinicalTrials.gov NCT06640400

## Introduction

Coronary artery disease (CAD) is caused by atherosclerotic lesions in the coronary arteries, resulting in narrowing or blockage of the lumens of the vessels; this, in turn, causes myocardial ischemia, hypoxia, and necrosis [1]. CAD has a high rate of morbidity and mortality and poses a severe threat to human health [2]. Percutaneous coronary intervention (PCI) uses cardiac catheterization to improve myocardial perfusion by unblocking narrowed or occluded coronary arteries [3]. As the least invasive revascularization procedure available, PCI has become the mainstay of treatment for CAD [4]. However, as an invasive test and treatment method, it is often accompanied by a variety of postprocedural complications, including in-stent thrombosis or restenosis, no or slow reflow, arrhythmia, and an inflammatory response, which can significantly affect a patient’s condition and prognosis [5]. Treatments for common complications after PCI include pharmacologic therapy, interventional repeat angioplasty, or stent implantation [5]. However, medication can also lead to adverse effects, and such interventions as well as surgery are risky and costly [6]. Therefore, there is new interest in the exploration of noninvasive, low-side-effect treatments to prevent postoperative complications and promote cardiac rehabilitation after PCI.

Cardiovascular ultrasound therapy, an emerging therapeutic tool, has attracted a great deal of attention because it has many biological effects and is noninvasive [7]. Cardiovascular ultrasound therapy sends a mechanical force to the tissues; it has mechanical, cavitation, and low thermal effects, triggering reactions at the cellular and molecular levels [8]. Previous studies have shown that cardiovascular ultrasound therapy promotes the expression of vascular growth factors, inhibits inflammatory responses and oxidative stress, reduces the apoptosis of cardiac cardiomyocytes, improves the microcirculation in ischemic tissues (thereby attenuating ischemia-reperfusion injury), and helps to protect cardiac function [7,9–13]. In addition, cardiovascular ultrasound therapy has a good safety and tolerability profile [14,15]. However, there are no relevant clinical research data to confirm the validity of these therapeutic effects; therefore, further studies are needed to determine the future role of cardiovascular ultrasound therapy in clinical use.

In order to clarify these issues, we designed a cardiovascular ultrasound treatment regimen for patients who had undergone PCI for CAD. In other words, we planned to conduct a multicenter, parallel-group, randomized controlled clinical trial to evaluate the rehabilitative efficacy of cardiovascular ultrasound therapy.

## Methods

### Aims of the study

Our study is intended to evaluate the rehabilitative efficacy of cardiovascular ultrasound therapy in patients with CAD who had already undergone PCI. Specifically, the study aims to examine the effects of cardiovascular ultrasound therapy on inflammatory markers, serum lipids, endothelial function, cardiac function, carotid artery plaque, and hemodynamics. In addition, the study will analyze patients’ heart rate variability (HRV), record their symptoms, and score depression and anxiety.

### Study design and ethical approval

This blinded, multicenter, parallel-group, randomized (1:1) controlled study will be conducted in 11 large hospitals in China, including Qilu Hospital of Shandong University, Qingdao Municipal Hospital, Yantai Yuhuanding Hospital, Linyi People’s Hospital, Tengzhou Central People’s Hospital, Weifang People’s Hospital, Jining No.1 People’s Hospital, Weihai Central Hospital, Liaocheng People’s Hospital, Dongying People’s Hospital, and Qilu Hospital of Shandong University (Qingdao). These hospitals were selected because of their significant experience and ability to recruit for PCI in cardiology as well as their ability to collaborate across disciplines to ensure that patients receive comprehensive treatment and rehabilitative support. The study protocol was reviewed and approved by the Ethics Committee of Qilu Hospital of Shandong University and other participating institutions (Ethics Committee reference number KYLL-202308-006). The study was registered on ClinicalTrials.gov, ID: NCT06640400.

Participants in this study will be recruited from the aforementioned medical institutions. Inclusion criteria call for hospitalized patients diagnosed (through laboratory and imaging tests) with CAD requiring PCI [16]. Following consent from the patients or their family members, screening procedures will be conducted by medical professionals to ensure compliance with the eligibility criteria. Only those individuals meeting all recruitment criteria and no exclusion criteria will be included in the study. Participant recruitment is expected to be completed by September 2026, data collection will be completed by December 2026, and, the authors estimate that cardiovascular ultrasound therapy may become a new modality in the treatment of post-PCI CAD rehabilitation. The study results will be published in a peer-reviewed journal in 2027.

### Participants: Inclusion and exclusion criteria

Participants will be recruited according to the following inclusion criteria:

- Age at enrollment: 18 years or older
- Confirmed CAD requiring elective PCI
- Thrombolysis in myocardial infarction (TIMI) flow grade 2 or above after PCI
- No intraoperative complications following PCI, such as entrapment, reflux, or perforation of the coronary artery
- Intact skin in the anterior chest area
- Cardiac enzymes and troponin I within normal range

Those meeting any of the following criteria are to be excluded from the study:

- ST-segment elevation myocardial infarction, non–ST-segment elevation myocardial infarction
- Occlusion of branch vessels during PCI
- Perioperative use of hormones or immunosuppressants
- Combined infection or other inflammatory diseases
- Postoperative fever
- Allergy to contrast media or cardiovascular ultrasound acoustic head–related materials
- Changes in lipid-lowering, antiplatelet, and antihypertensive drug regimens during treatment
- A clear diagnosis of autoimmune disease
- A history of other surgeries or trauma in the preceding 7 days
- Infection with the novel coronavirus during the preceding month
- Previous PCI treatment
- Previous cardiovascular ultrasound treatment
- Diabetes mellitus

### Calculation of sample size

Our calculations of sample size and power are based on the primary outcomes, utilizing a two-sided analysis with a statistical power of 90% (1 – β = .90) and a significance level of α = .05. The design and analysis of this study did not have sufficient references to accurately estimate the sample size. However, in 2023, we conducted the results of a preliminary study in patients with angina pectoris from coronary heart disease, with hsCRP levels from baseline to 20 ultrasound treatments as the primary outcome measure. It is estimated that a sample size of 63 participants is required for each group to have a 90% probability of demonstrating a statistically significant difference of 5% between groups for 20 sessions of cardiovascular ultrasound therapy (two-tailed test). Sample size estimation was performed using the PASS 15.0.5 procedure. To ensure that the study objectives were statistically competent and robust, we included 200 patients. To validate the above estimates, when the collection of 10 patients per group is complete, a comparison of the primary endpoint between groups will be performed, and using the results of the analysis, a sample size estimate will be performed to determine the final size of the study sample, as this strategy has been suggested by a relevant review of sample size estimation methods [17,18].

### Randomization and blinding

#### Sequence generation and concealment mechanism.

In this study, stratified block randomization was employed, divided into 10 tiers according to the study center, with 20 patients in each group, to ensure a balanced number of cases between groups. Within the stratum, participants were divided into two groups using a randomized 1:1 ratio, participants will be allocated into either of two groups: the cardiovascular ultrasound therapy group and the control group. This process will be facilitated by a computer-generated randomization sequence. To uphold the integrity of the allocation process, a team of research assistants not involved in clinical care or outcome assessment will meticulously craft sequentially numbered, opaque, sealed envelopes based on the predefined randomization list. This meticulous approach will ensure the safeguarding of confidentiality and the autonomy of allocation data. When appropriate, the assistants will open envelopes and ensure the coordination of therapeutic interventions.

To ensure the reliability and accuracy of the study results, we use blinding, the assignment results will be concealed from the attending physicians, the patients and the investigative team members responsible for the collection of primary outcome data will remain masked. Consequently, the study design adheres to the framework of a blinded controlled trial. Patients will retain the prerogative to decline study participation, in which case they would receive standard care only.

#### Who will take informed consent?

The research assistants (CHs) will take informed consent before trial baseline evaluation in the participants’ reception room. Participants will be told why the study is being conducted, what they will be doing, and the possible benefits and risks. If participants have any questions, they will be free to ask them. Then, being fully informed, they will be able to decide whether or not to participate in the study.

#### Additional consent provisions for the collection and use of participant data and biological specimens.

No additional biological samples have been collected for the study. No subject data are currently available for future studies. Additional consent from participants will be obtained if relevant data should be required for future studies.

### Interventions

#### Explanation for the choice of comparators.

We intend to select the prevailing standard (sham stimulating) allopathic therapy as the control condition to determine if the expected benefits of cardiovascular ultrasound therapy will produce better anti-inflammatory results than standard therapy.

#### Intervention description.

Before treatment, all participants will attend a comprehensive health education class conducted by a research assistant and lasting for about 20 minutes. It would include essential topics such as CAD, risk factors, clinical manifestations, treatment interventions, and a detailed discussion of the evidence-based efficacy and safety aspects of cardiovascular ultrasound therapy. Moreover, patients will be advised to adhere to a light diet, refrain from smoking and excessive alcohol consumption, engage in moderate physical activity, maintain regular medical checkups, and diligently follow the recommendations of their healthcare providers.

Participants allocated to the intervention group will undergo a 14-day regimen utilizing a medical cardiovascular ultrasound therapy device (838C-M-L-I/II, Shenzhen, China). The ultrasound instrument is equipped with a sound head comprising five transducer units operating at an ultrasonic frequency of 0.84 MHz with a sound intensity range of 1 W/cm^2^–1.25 W/cm^2^. The therapeutic ultrasound sessions require a controlled environmental temperature. Patients will assume the supine position, exposing the precordial region, with the 5-pronged head positioned parallel to the heart’s long axis; it should cover the entire precordial region including the right and left coronary arterial trunks and the aortic root. The five transducer units function sequentially in a scanning pattern, with each unit operating for 5 seconds and transitioning with a 0.5-second interval, following a clockwise rotation until the treatment has been completed. The cardiovascular ultrasound therapy device operates in a pulsed mode, and each treatment session lasts for 20 minutes; there would be two daily sessions for a total of 20 treatments.

Participants in the control group will not receive a real treatment with cardiovascular ultrasound therapy, but will be treated with a simulator (sham stimulating). Cardiovascular ultrasound therapy will be assessed at baseline (24th-hour post-PCI), on the 14th day, and at the end of the first and third months post-intervention.

#### Criteria for discontinuing or modifying allocated interventions.

Upon request and at any point, patients are free to withdraw from participation without adverse consequences, and thereafter their data would neither be collected nor utilized for analysis. Cardiovascular ultrasound therapy is a safe treatment modality that has been approved by the China Food and Drug Administration. To date, no serious adverse events have been reported with the use of cardiovascular ultrasound therapy for cardiovascular disease. Nevertheless, our nursing staff will vigilantly monitor possible mild reactions such as local swelling, minor bleeding, heightened local pain sensations, and local hyperesthesia or reduced sensitivity. If such reactions should occur, treatment will be administered promptly. Participants may discontinue their involvement in the study should they encounter adverse events that they find unacceptable.

#### Strategies to improve adherence to interventions.

To promote adherence to the interventions, free measures (including vascular endothelial function testing, hemodynamic monitoring, the 6-minute walk test [6MWT], HRV testing, recording of symptoms, and scoring of depression and anxiety) will be offered following treatment. We will also be offering financial incentives or free post-study services to encourage continued participation. Additionally, implementing strategies like follow-up phone calls or sending regular reminders could promote long-term adherence to the study’s timeline.

#### Relevant concomitant care permitted or prohibited during the trial.

All participants will receive routine treatment but no additional cardiac or other rehabilitation.

#### Provisions for posttrial care.

The trial interventions have been meticulously designed to ensure minimal to no harm to participants. However, should a participant sustain an injury during the trial, we would promptly assess, document, and treat the injury while also covering all associated medical expenses.

### Outcomes and measurements

#### Primary outcome.

The primary outcome measures will be the levels of hsCRP in the peripheral serum following 20 cardiovascular ultrasound therapy sessions. Comparative analyses will be conducted between the cardiovascular ultrasound therapy intervention and control groups along with pre- and postintervention assessments.

#### Secondary outcome.

***Indicators of serum myocardial injury and blood lipid levels*:** Serum biomarkers indicative of myocardial injury encompass creatine kinase isoenzymes (CK-MB), cardiac troponin I (c-TnI), and myeloperoxidase (MPO). Lipid profile markers in serum comprise total cholesterol (TC), triglycerides (TG), low-density lipoprotein cholesterol (LDL-c), high-density lipoprotein cholesterol (HDL-c), apolipoprotein A (ApoA), apolipoprotein B (ApoB), lipoprotein (a), and oxidized low-density lipoprotein (ox-LDL) in the peripheral blood.

Monitoring of serum biomarkers indicative of myocardial injury will be conducted at baseline (24th hour post PCI), on the 14th day, and at the end of the 1st and 3rd months post intervention.

***Serum markers of endothelial function*:** The serum markers utilized to assess endothelial function encompass endothelial nitric oxide synthase (eNOS), endothelin-1 (ET-1), and vascular endothelial growth factor (VEGF) in the peripheral blood.

Monitoring of serum endothelial function will be conducted at baseline (24th hour post PCI); on the 7th and 14th days; and at the end of the 1st and 3rd months post intervention.

***Serum inflammatory factors*:** Each morning, 5 mL of fasting venous blood will be drawn by nursing staff to assess the proportion of inflammatory cells and measure levels of inflammatory markers in the peripheral blood.

Monitoring of serum inflammatory factors will be conducted at baseline (24th hour post PCI), on the 14th day, and at the end of the 1st and 3rd months post intervention.

***Hemodynamic parameters*:** These encompass a comprehensive range of indicators, including cardiac index (CI), cardiac output (CO), stroke volume (SV), stroke volume index (SVI), mean arterial pressure (MAP), stroke systemic vascular resistance index (SSVRI), left ventricular stroke work index (LVSWI), systemic vascular resistance index (SVRI), ejection phase contraction index (EPCI), inotropic state index (ISI), systemic vascular resistance (SVR), vascular resistance (VR), and mean heart rate (mHR).

Certified sonographers will monitor hemodynamic parameters at baseline (24th hour post PCI); on the 7th and 14th days; and at the end of the 1st and 3rd months post intervention.

***Echocardiography*:** The echocardiogram parameters will encompass E/e’ ratio, left ventricular ejection fraction (LVEF), and wall thickening fraction (WTF). WTF is defined as [(end-diastolic wall thickness − end-systolic wall thickness)/ end-diastolic wall thickness] × 100 (%).

Echocardiographic assessments will be conducted by certified sonographers proficient in thoracic echocardiography. This will be done at multiple time points: at baseline (24th hour post PCI), on the 7th and 14th days, and at the end of the 1st and 3rd months post intervention.

***Ultrasound examination of carotid plaques*:** Ultrasonography of carotid plaques includes their location, size (length x width), and morphology as well as the intima-media thickness (IMT) and Crouse’s score of the common carotid artery. IMT was measured at 1.5 cm from the origin, branch, trunk, and bifurcation of the common carotid artery bilaterally. During plaque examination, plaque thickness was recorded and Crouse’s score was calculated by adding the thickness of each plaque. Common carotid artery hemodynamic indexes, including the peak systolic velocity (PSV), end diastolic velocity (EDV), and resistance index (RI) of the common carotid artery were detected.

A certified sonographer proficient in examining the carotid arteries will perform ultrasound evaluations at multiple time points: at baseline (24th hour post PCI); on the 7th and 14th days; and at the end of the 1st and 3rd months post intervention.

***Six-minute walk test*:** The 6MWT quantifies the maximal distance an individual can walk in 6 minutes, serving as an indicator of the patient’s exercise capacity and cardiorespiratory fitness during routine physical activities.

A certified cardiologist will conduct the 6MWT at various time points, including at enrollment, on the14th day, and at the end of the 1st and 3rd months post intervention.

***Short-term heart rate variability*:** HRV refers to the fluctuation in the timing of consecutive heartbeats or the variance in heart rate. It is determined by the duration between two successive R-R intervals, representing the slight deviation between each cardiac cycle. HRV assessment will be conducted using the multiparameter monitor (WMMS201, Beijing, China). With the patient at rest and in a supine position, short-term (5-minute) HRV will be measured during daytime hours [19].

Certified cardiologists will perform short-term HRV assessments at baseline (24th hour post PCI), on the 7th and 14th days, and at the end of the 1st and 3rd months post intervention.

***Tools for mental health assessment*:** The Patient Health Questionnaire-9 (PHQ-9) is a critical instrument for the screening, diagnosis, and assessment of depression, enabling the measurement of its severity (S1 Table). Concurrently, the 7-item Generalized Anxiety Disorder Scale (GAD-7) enables the evaluation of generalized anxiety disorder (S2 Table). Similarly, the Pittsburgh Sleep Quality Index (PSQI) stands as a widely recognized assessment tool for determining sleep quality (S3 Table), and the Seattle Angina Questionnaire (SAQ) functions as a self-administered assessment tool designed to evaluate specific functional status and quality of life among individuals with CAD (S4 Table).

Mental health assessments will be conducted by certified psychologists at various time points: at baseline (24th hour post PCI), on the 14th day, and at the end of the 1st and 3rd months post intervention.

**Fig 1:**
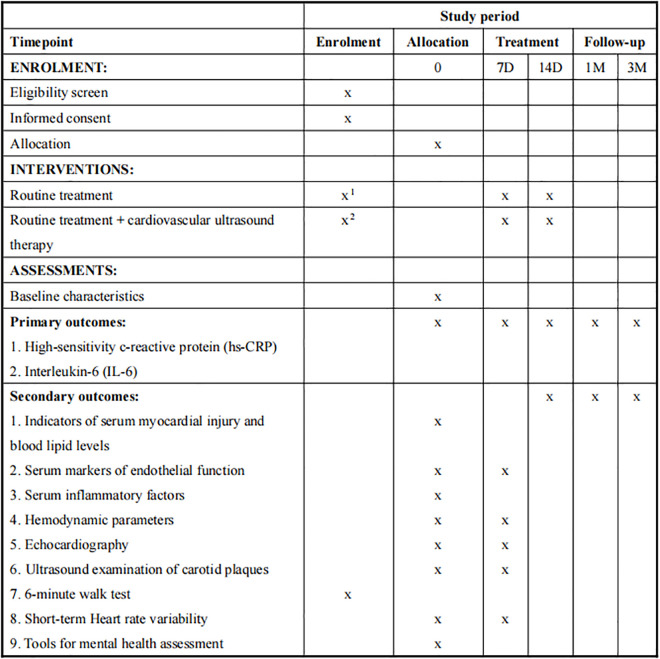
SPIRIT schedule of enrolment, interventions, and follow-up of the study. The specific time points are listed in the columns and the events are given in the rows. Participants will be randomly allocated to Routine treatment (x^1^) or Routine treatment with cardiovascular ultrasound therapy (x^2^). All participants will have a follow-up visit at 1 months, 3 months. D, day; M, month. Tools for mental health assessment would include recording and scoring the patient’s Health Questionnaire-9, 7-item Generalized Anxiety Disorder Scale, Pittsburgh Sleep Quality Index, and Seattle Angina Questionnaire.

***Participant timeline*:** This study is scheduled to begin in 2025. The participant timeline is shown in Fig 1.

### Data collection and management

#### Plans for assessment and collection of outcomes.

The primary outcome data will be captured both at the baseline and the conclusion of treatment (20th time); the secondary outcome data will be gathered during the baseline assessment (24th hour post PCI); on the 7th and 14th days; and at the end of the 1st and 3rd months post intervention. Every variable outlined in the protocol will be meticulously documented within an encrypted Microsoft Office Excel spreadsheet. The investigators entering the data into the spreadsheet will be responsible for ensuring the accuracy and comprehensiveness of all the information. A day-to-day management group comprising the principal investigator, a doctoral student, a research coordinator, and an assistant manager will oversee these procedures.

#### Plans to promote participant retention and completion of follow‑up.

All evaluations and treatments will be conducted during the patients’ hospital stay. Results regarding vascular endothelial function, hemodynamic monitoring, HRV, 6MWTs, and any trial-related score administered before or after treatment will be offered to the patients at no cost. If a patient opts to withdraw from the study prematurely (within the 14-day treatment period), their data will not be included in the final analysis. Maintaining patient engagement throughout the planned outcome assessments is crucial, and every effort should be made to promote patient retention for the entire 14 days.

#### Data management.

Initially, investigators will collect data on participants’ baseline status; then, after randomization, data will be collected daily. All acquired data will be securely stored electronically in a confidential database with restricted access. To ensure security, the transfer process will include encryption, and any personally identifiable information will be expunged. Reversal of the encryption (i.e., identification of the data) will be permitted only during the concluding phase of the trial. Participants who choose not to continue or are found to meet any of the exclusion criteria will be withdrawn from the study. The suspension details, including the date and reason, will be documented and the participants’ data will not be retained.

#### Confidentiality.

Each participant’s data will be given a unique identifier and securely stored on a dedicated server accessible only to authorized researchers with administrative privileges. After the trial, only data without personal information will remain accessible.

### Statistical analysis

#### Determination of primary and secondary outcomes.

The baseline characteristics of both groups will be summarized using appropriate descriptive statistics. Analysis of both primary and secondary outcomes will be based on the intention-to-treat approach, ensuring that all participants remain assigned to their original group throughout the study.

Normally distributed data will be presented as the mean (standard deviation), and non–normally distributed data will be depicted as the median (interquartile range). Group comparisons will be conducted using independent sample tests, and within-group comparisons before and after treatment will be evaluated using paired sample tests. Clinical symptom variables will be portrayed as percentages. Categorical data will be assessed using either the chi-squared test or Fisher’s exact test. A significance level of.05 will be employed to interpret the *P* values. The statistical analysis will be conducted utilizing SPSS version 26 statistical software (IBM, Armonk, NY).

#### Interim analyses.

No interim analyses are being planned.

#### Methods for additional analyses (e.g., subgroup analyses).

No subgroup analyses will be conducted.

#### Analytic methods for handling protocol nonadherence and statistical methods for handling missing data.

We anticipate minimal missing data (<10%) for the primary outcome given that all participants will be in patients with regular daily contact maintained during treatment. The evaluation of missing data effects will involve comparing differences under plausible and extreme missing data scenarios. In the event of a significant primary test result, we will also assess the interaction size between group assignment and missing data necessary to render the difference nonsignificant. Should 10% or more of the baseline samples be excluded due to missing data, we will apply multiple imputation techniques for comprehensive analysis.

#### Plans to give access to the full protocol, participant‑level data, and statistical code.

Anonymized participant data can be made available upon a reasonable request from the corresponding author. However, there are no plans to provide public access to participant data or the statistical code.

#### Oversight and monitoring.

***Composition of the coordinating center and trial steering committee*:** Ongoing clinical monitoring will be overseen by a day-to-day management team comprising the principal investigator (PI), a doctoral student, a research coordinator, and an assistant manager. Regular weekly meetings will be held by the group to ensure effective coordination and management of the study. This trial does not have a trial steering committee.

***Composition of the data monitoring committee—Its role and reporting structure*:** We will establish a formal data monitoring committee composed of experts in the cardiovascular field, statistical analysis and ethics to regularly review the data to ensure quality, independently determine adverse events and supervise rectification to ensure the integrity and accuracy of the data.

***Adverse events reporting and harms*:** All adverse events, defined as any unfavorable or unintended reactions to the intervention, will be meticulously documented based on patient-reported symptoms and observational assessments during each visit. To date, no adverse events have been reported with use of the cardiovascular ultrasound therapy regimen in cardiovascular disease. Potential mild adverse reactions**—**including mild local swelling, heightened local pain response, and altered local sensitivity**—**will be monitored closely. In the event of such symptoms, prompt and suitable symptomatic treatment will be administered.

***Frequency and plans for auditing trial conduct*:** Not applicable. No auditing is planned in this trial. The PI will meet weekly during the study period to review trial progress.

***Plans for communicating important protocol amendments to relevant parties (e.g., trial participants, ethical committees)***: Any proposed modifications to the study protocol require prior approval from the Ethics Committee of Qilu Hospital of Shandong University. Upon approval, these protocol amendments must be documented in the trial register and, for transparency and accuracy, integrated into the final research report.

***Dissemination plans*:** The outcomes of the clinical trial are intended for dissemination through publication in medical journals and presentations at national and international conferences. The lead and corresponding authors will be responsible for overseeing this process.

## Discussion

**Fig 2:**
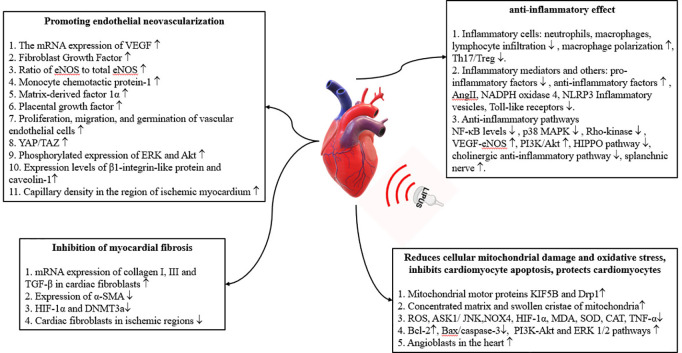
Mechanism of action of cardiovascular ultrasound therapy [24–31]. Abbreviations: VEGF, vascular endothelial growth factor; miRNA, micro RNA; NF-B, nuclear factor B; NO, nitric oxide; PI3K-Akt, phosphatidylinositol 3-kinase-protein kinase B; a-SMA, a-smooth muscle actin; TGF-β, transforming growth factor β; HIF-1α, hypoxia inducible factor-1α; DNMT3a, DNA methyltransferase 3a; KIF5B, kinesin superfamily of motor protein 5B; Drp1, dynamin-related protein 1; ROS, reactive oxygen species; ASK1, apoptosis signal-regulated kinase 1; JNK, c-JunN-terminal kinase; NOX4, NADPH oxidase 4; MDA, malondialdehyde; SOD, superoxide dismutase; CAT, catalase; TNF-α, tumor necrosis factor alpha; Bax, bcl-2 family of pro-apoptotic factors; caspase-3, cysteine protease-3; ERK 1/2, extracellular regulated protein kinase 1/2.

As far as we know, at its conclusion, this study will be the first randomized controlled investigation into the rehabilitative efficacy of cardiovascular ultrasound therapy in individuals with CAD post PCI, thus adding a new dimension to the existing literature. Although prior research has examined LIPUS’s (Low-intensity pulsed ultrasound) efficacy and safety in refractory angina and other vascular disorders, clinical studies in patients undergoing PCI for CAD are notably scarce [14,20,21]. Earlier cell biology and animal experiments have established the LIPUS’s potential for promoting the regeneration of vascular endothelial cells and initiating angiogenesis. This modality has also exhibited anti-inflammatory properties, attenuated mitochondrial damage, reduced oxidative stress, inhibited cardiomyocyte apoptosis, and safeguarded against myocardial remodeling [11,22,23] (Fig 2). Therefore, a randomized controlled trial is needed to study the effect of cardiovascular ultrasound therapy on cardiac health, endothelial function, inflammation levels, and hemodynamics in post-PCI CAD patients. The publication of such a study’s protocols should help to bridge existing gaps in knowledge and provide guidance for future studies.

Finally, we acknowledge that our study protocol has several limitations. First, our comparative analysis contrasts cardiovascular ultrasound therapy only with nontreatment; thus there can be no direct comparison with standard clinical anti-inflammatory, immunomodulatory, or anticoagulation therapies. Thus the comprehensiveness of our observations regarding clinical efficacy must be limited. Second, the geographically confined nature of this study (being conducted only in China) might constrain the generalizability of the results to other populations. However, it reduces the differences in treatment between different countries and decreases confounding biases. Moreover, the inherent challenge of blinding when cardiovascular ultrasound therapy equipment is being employed requires consideration, thus posing a methodologic limitation yet to be addressed. After completing the research and obtaining safety data, we will consider conducting a three-group controlled study (cardiovascular ultrasound, placebo, and standard treatment) to provide a template for subsequent multinational trials.

## Supporting information

S1 Checklist SPIRIT 2013 checklist: Recommended items to address in a clinical trial protocol and related document. (PDF)

S2 File The copy of research protocol approved by the ethics committee. A copy of the study protocol of the rehabilitative efficacy of cardiovascular ultrasound therapy after percutaneous coronary intervention in patients with coronary artery disease: A multicenter, parallel-group, randomized controlled study.
(DOCX)

S3 File The English translation of the ethics approval document. (DOCX)

S1 Table The Patient Health Questionnaire (PHQ-9). (DOCX)

S2 Table Generalized Anxiety Disorder 7-item (GAD-7) scale. (DOCX)

S3 Table Pittsburgh Sleep Quality Index (PSQI). (DOCX)

S4 Table The Seattle Angina Questionnaire-7. (DOCX)

## Abbreviations

CHD: Coronary heart disease
LIPUS: Low-intensity pulsed ultrasound
PCI: Percutaneous coronary intervention
CAD: Coronary artery disease
CFDA: China Food and Drug Administration
hs-CRP: high-sensitivity c-reactive protein
IL-6: Interleukin-6
CK-MB: Creatine kinase isoenzymes
c-TnI: Cardiac troponin I
MPO: Myeloperoxidase
TC: Total cholesterol
TG: Triglycerides
LDL-c: Low-density lipoprotein cholesterol
HDL-c: High-density lipoprotein cholesterol
ApoA: Apolipoprotein A
ApoB: Apolipoprotein B
ox-LDL: oxidized low-density lipoprotein
eNOS: endothelial nitric oxide synthase
ET-1: Endothelin-1
VEGF: Vascular endothelial growth factor
PCT: Procalcitonin
CI: Cardiac index
CO: Cardiac output
SV: Stroke volume
SVI: Stroke volume index
MAP: mean arterial pressure
SSVRI: Stroke systemic vascular resistance index
LVSWI: Left ventricular stroke work index
SVRI: Systemic vascular resistance index
EPCI: Ejection phase contraction index
ISI: Inotropic state index
SVR: Systemic vascular resistance
VR: Vascular resistance
mHR: mean heart rate
LVEF: Left ventricular ejection fraction
WTF: Wall thickening fraction
6MWT: 6-minute walk test
IMT: intima-media thickness
PSV: peak systolic velocity
EDV: end diastolic velocity
RI: resistance index
PHQ-9: Patient Health Questionnaire-9
GAD-7: 7-item Generalized Anxiety Disorder Scale
PSQI: Pittsburgh Sleep Quality Index
SAQ: Seattle Angina Questionnaire
HRV: Heart rate variability
