# Supplementary material for: A study protocol of the rehabilitative efficacy of cardiovascular ultrasound therapy after percutaneous coronary intervention in patients with coronary artery disease: A multicenter, parallel-group, randomized controlled study
Source: PLoS One. 2025 Oct 16;20(10):e0327557. doi: 10.1371/journal.pone.0327557 (PMC12530608; doi:10.1371/journal.pone.0327557)
Supplement: S2 File — A copy of the study protocol of the rehabilitative efficacy of cardiovascular ultrasound therapy after percutaneous coronary intervention in patients with coronary artery disease: A multicenter, parallel-group, randomized controlled study. (DOCX) [file pone.0327557.s002.docx]

**Project Title: A study protocol of the rehabilitative efficacy of cardiovascular ultrasound therapy after percutaneous coronary intervention in patients with coronary artery disease: A multicenter, parallel-group, randomized controlled study**

| **Project Leader** | Lin Shen |
| --- | --- |
| **Project Leader** | Cheeloo College of Medicine, Shandong University |
| **Project Contact** | Jie Peng |
| **Project Participants** | **Head of Project Participation Unit** |
| Qilu Hospital of Shandong University | Lin Shen |
| Qingdao Municipal Hospital | Yibing Shao |
| Yantai Yuhuangding Hospital | Lin Zhong |
| Linyi People's Hospital | Yanjin Wei |
| Tengzhou Central People's Hospital | Jingpeng Yan |
| Weifang People's Hospital | Chuanliang Liu |
| Jining No.1 People's Hospital | Changjie Ren |
| Weihai Central Hospital | Jianhua Zhou |
| Liaocheng People’s Hospital | Shuqin Liu |
| Dongying People’s Hospital | Fenglei Zhang |
| Qilu Hospital of Shandong University (Qingdao) | Beian You |
| **Statistical analysis unit** | Qilu Hospital of Shandong University |

Note: The investigator grants permission to publish this protocol if my work is accepted.

**Summary of Study Protocol**

| **Project Name** | A study protocol of the rehabilitative efficacy of cardiovascular ultrasound therapy after percutaneous coronary intervention in patients with coronary artery disease: A multicenter, parallel-group, randomized controlled study |
| --- | --- |
| **Objective** | The aim of this study is intended to evaluate the rehabilitative efficacy of cardiovascular ultrasound therapy in patients with coronary artery disease (CAD) who had already undergone percutaneous coronary intervention (PCI). Specifically, the study aims to examine the effects of cardiovascular ultrasound therapy on inflammatory markers, serum lipids, endothelial function, cardiac function, carotid artery plaque, and hemodynamics. In addition, the study will analyze patients’ heart rate variability (HRV), record their symptoms, and score depression and anxiety. |
| **Research Hypothesis** | Cardiovascular ultrasound therapy has the potential to reduce perioperative myocardial damage, reduce the production of inflammatory factors and inflammatory cells, and has a certain regulatory effect on blood lipids, platelet function, hemodynamics and blood pressure in patients after PCI. |
| **Study Design** | This study is a multicenter, parallel-group, randomized controlled clinical trial. Subjects were enrolled by randomization into groups of 20 per center, with 10 in the control group and 10 in the treatment group. Each participating unit completes the relevant content of the subject case list. |
| **Subject population** | **Inclusion Criteria:**  • Age at enrollment: 18 years or older;  • Confirmed CAD requiring elective PCI;  • Thrombolysis in myocardial infarction (TIMI) flow grade 2 or above after PCI;  • No intraoperative complications following PCI, such as entrapment, reflux, or perforation of the coronary artery;  • Intact skin in the anterior chest area;  • Cardiac enzymes and troponin I within normal range.  **Exclusion Criteria:**  • ST-segment elevation myocardial infarction and non–ST-segment elevation myocardial infarction;  • Occlusion of branch vessels during PCI;  • Perioperative use of hormones or immunosuppressants;  • Combined infection or other inflammatory diseases;  • Postoperative fever;  • Allergy to contrast media or cardiovascular ultrasound acoustic head–related materials;  • Changes in lipid-lowering, antiplatelet, and antihypertensive drug regimens during treatment;  • A clear diagnosis of autoimmune disease;  • A history of other surgeries or trauma in the preceding 7 days;  • Infection with the novel coronavirus during the preceding month;  • Previous PCI treatment;  • Previous cardiovascular ultrasound treatment;  • Diabetes mellitus. |
| **Diagnostic criteria** | The diagnosis of coronary heart disease refers to the diagnostic criteria of the American Heart Association, and the lumen diameter of at least one or more major coronary arteries is confirmed by coronary angiography to be narrowed by ≥50%, combined with medical history, electrocardiogram and coronary CTA. |
| **Sample size** | The sample size of the intervention group and the control group was calculated using PASS 15 software. Our calculations of sample size and power are based on the primary outcomes, utilizing a two-sided analysis with a statistical power of 90% (1 – β = 0.90) and a significance level of α = 0.05. Owing to the lack of prior studies examining cardiovascular ultrasound therapy following PCI, the sample size was determined following an extensive evaluation by a panel of experts. Targeting long-term inflammation and cardiovascular efficacy, they selected high-sensitivity C-reactive protein (hsCRP) as the primary biomarker, given its relevance and the larger sample size required by interleukin 6. Cardiovascular ultrasound is estimated to reduce hsCRP levels by an average of 4 mg/L. To meet this target, 160 participants (80 per group in a 1:1 allocation) are projected to be needed. Factoring in an anticipated dropout rate of 20%, the final sample size would be adjusted to 200 participants (100 per group), ensuring adequate statistical power and robustness for the study’s objectives. |
| **Study outcomes** | 1. **Primary** **outcome**   The primary outcome measures will focus on the levels of hsCRP and interleukin-6 in the peripheral serum.   1. **Secondary outcome**   **1.1 Indicators of serum myocardial injury and blood lipid levels**  Serum biomarkers indicative of myocardial injury encompass creatine kinase isoenzymes (CK-MB), cardiac troponin I (c-TnI), and myeloperoxidase (MPO). Lipid profile markers in serum comprise total cholesterol (TC), triglycerides (TG), low-density lipoprotein cholesterol (LDL-c), high-density lipoprotein cholesterol (HDL-c), apolipoprotein A (ApoA), apolipoprotein B (ApoB), lipoprotein (a), and oxidized low-density lipoprotein (ox-LDL) in the peripheral blood.  **1.2 Serum markers of endothelial function**  The serum markers utilized to assess endothelial function encompass endothelial nitric oxide synthase (eNOS), endothelin-1 (ET-1), and vascular endothelial growth factor (VEGF) in the peripheral blood.  **1.3 Serum markers of endothelial function**  The serum markers utilized to assess endothelial function encompass endothelial nitric oxide synthase (eNOS), endothelin-1 (ET-1), and vascular endothelial growth factor (VEGF) in the peripheral blood.  **1.4 Serum inflammatory factors**  Each morning, 5 mL of fasting venous blood will be drawn by nursing staff to assess the proportion of inflammatory cells and measure levels of inflammatory markers in the peripheral blood.  **1.5 Hemodynamic parameters**  These encompass a comprehensive range of indicators, including cardiac index (CI), cardiac output (CO), stroke volume (SV), stroke volume index (SVI), mean arterial pressure (MAP), stroke systemic vascular resistance index (SSVRI), left ventricular stroke work index (LVSWI), systemic vascular resistance index (SVRI), ejection phase contraction index (EPCI), inotropic state index (ISI), systemic vascular resistance (SVR), vascular resistance (VR), and mean heart rate (mHR).  **1.6 Echocardiography**  The echocardiogram parameters will encompass E/e’ ratio, left ventricular ejection fraction (LVEF), and wall thickening fraction (WTF). WTF is defined as [(end-diastolic wall thickness − end-systolic wall thickness)/ end-diastolic wall thickness] × 100 (%).  **1.7 Ultrasound examination of carotid plaques**  Ultrasonography of carotid plaques includes their location, size (length x width), and morphology as well as the intima-media thickness (IMT) and Crouse’s score of the common carotid artery.  **1.8 Six-minute walk test**  The six-minute walk test (6MWT) quantifies the maximal distance an individual can walk in 6 minutes, serving as an indicator of the patient's exercise capacity and cardiorespiratory fitness during routine physical activities.  **1.9 Short-term heart rate variability**  HRV refers to the fluctuation in the timing of consecutive heartbeats or the variance in heart rate. It is determined by the duration between two successive R-R intervals, representing the slight deviation between each cardiac cycle. HRV assessment will be conducted using the amor HRV Guard Analyzer (ZSY-1, Taiwan, China). With the patient at rest and in a supine position, short-term (5-minute) HRV will be measured during daytime hours.  **2.0 Tools for mental health assessment**  The Patient Health Questionnaire-9 (PHQ-9) is a critical instrument for the screening, diagnosis, and assessment of depression, enabling the measurement of its severity. Concurrently, the 7-item Generalized Anxiety Disorder Scale (GAD-7) enables the evaluation of generalized anxiety disorder. Similarly, the Pittsburgh Sleep Quality Index (PSQI) stands as a widely recognized assessment tool for determining sleep quality, and the Seattle Angina Questionnaire (SAQ) functions as a self-administered assessment tool designed to evaluate specific functional status and quality of life among individuals with CAD. |
| **Follow-up plan** | The patient was followed up at the end of the 1st and 3rd months post intervention. |
| **Sample collection** | √ Yes □No  10 ml/person of venous blood should be collected each time, for a total of 50ml/person for 5 sessions. |
| **Non-routine inspection** | □Yes √ No |
| **Statistical methods** | The baseline characteristics of both groups will be summarized using appropriate descriptive statistics. Analysis of both primary and secondary outcomes will be based on the intention-to-treat approach, ensuring that all participants remain assigned to their original group throughout the study.  Normally distributed data will be presented as the mean (standard deviation), and non–normally distributed data will be depicted as the median (interquartile range). Group comparisons will be conducted using independent sample tests, and within-group comparisons before and after treatment will be evaluated using paired sample tests. Clinical symptom variables will be portrayed as percentages. Categorical data will be assessed using either the chi-squared test or Fisher’s exact test. A significance level of.05 will be employed to interpret the P values. The statistical analysis will be conducted utilizing SPSS version 26 statistical software (IBM, Armonk, NY). |
| **Research risk self-assessment** | □ High-risk research □ Medium risk research √ Low-risk research  The group conducted relevant clinical studies at an early stage with positive results and no adverse effects. Previous studies have demonstrated the safety of cardiovascular ultrasound treatment modalities. The clinical trial is a multi-center clinical study with a large sample size, standardized design, high feasibility and low risk. |
| **Research Funding** | □ Fully funded by:  √ Partially funded by: Shenzhen Shengxiang High-tech Co., Ltd.  □ Nofunding  The research group signs horizontal project contracts with cooperative enterprises, and provides financial support on a regular basis according to the contracts. |
| **Corporate involvement** | □ No √ Yes  Company name: Shenzhen Shengxiang High Technology Co., Ltd  Company contact person: Zhiming Ning.  Enterprise contact to undertake the content: Technical information related to extracorporeal ultrasound therapy; 1 set of extracorporeal ultrasound therapy equipment; All consumables for extracorporeal ultrasound therapy required for the test. |
| **Other notes** | NO |

**Detailed study of the reference frame of the program**

**1 Introduction**

Coronary artery disease (CAD) is caused by atherosclerotic lesions in the coronary arteries, resulting in narrowing or blockage of the lumens of the vessels; this, in turn, causes myocardial ischemia, hypoxia, and necrosis ^[1]^. CAD has a high rate of morbidity and mortality and poses a severe threat to human health ^[2]^. Percutaneous coronary intervention (PCI) uses cardiac catheterization to improve myocardial perfusion by unblocking narrowed or occluded coronary arteries ^[3]^. As the least invasive revascularization procedure available, PCI has become the mainstay of treatment for CAD ^[4]^. However, as an invasive test and treatment method, it is often accompanied by a variety of postprocedural complications, including in-stent thrombosis or restenosis, no or slow reflow, arrhythmia, and an inflammatory response, which can significantly affect a patient’s condition and prognosis ^[5]^. Treatments for common complications after PCI include pharmacologic therapy, interventional repeat angioplasty, or stent implantation ^[5]^. However, medication can also lead to adverse effects, and such interventions as well as surgery are risky and costly ^[6]^. Therefore, there is new interest in the exploration of noninvasive, low-side-effect treatments to prevent postoperative complications and promote cardiac rehabilitation after PCI.

Cardiovascular ultrasound therapy cardiovascular ultrasound therapy, an emerging therapeutic tool, has attracted a great deal of attention because it has many biological effects and is noninvasive ^[7]^. Cardiovascular ultrasound therapy sends a mechanical force to the tissues; it has mechanical, cavitation, and low thermal effects, triggering reactions at the cellular and molecular levels ^[8]^. Previous studies have shown that cardiovascular ultrasound therapy promotes the expression of vascular growth factors, inhibits inflammatory responses and oxidative stress, reduces the apoptosis of cardiac cardiomyocytes, improves the microcirculation in ischemic tissues (thereby attenuating ischemia-reperfusion injury), and helps to protect cardiac function ^[7,9-13]^. In addition, cardiovascular ultrasound therapy has a good safety and tolerability profile ^[14,15]^. However, there are no relevant clinical research data to confirm the validity of these therapeutic effects; therefore, further studies are needed to determine the future role of cardiovascular ultrasound therapy in clinical use.

In order to clarify these issues, we designed a cardiovascular ultrasound treatment regimen for patients who had undergone PCI for CAD. In other words, we planned to conduct a multicenter, parallel-group, randomized controlled clinical trial to evaluate the rehabilitative efficacy of cardiovascular ultrasound therapy.

**2 Aims of the study**

Our study is intended to evaluate the rehabilitative efficacy of cardiovascular ultrasound therapy in patients with coronary artery disease (CAD) who had already undergone percutaneous coronary intervention (PCI). Specifically, the study aims to examine the effects of cardiovascular ultrasound therapy on inflammatory markers, serum lipids, endothelial function, cardiac function, carotid artery plaque, and hemodynamics. In addition, the study will analyze patients’ heart rate variability (HRV), record their symptoms, and score depression and anxiety.

**3 Study program**

**3.1 Study design and ethical approval**

This nonblinded, multicenter, parallel-group, randomized (1:1) controlled study will be conducted in 10 large hospitals in China, including Qilu Hospital of Shandong University, Qingdao Municipal Hospital, Yantai Yuhuanding Hospital, Linyi People’s Hospital, Tengzhou Central People’s Hospital, Weifang People’s Hospital, Jining No.1 People’s Hospital, Weihai Central Hospital, Liaocheng People’s Hospital, Dongying People’s Hospital, and Qilu Hospital of Shandong University (Qingdao). These hospitals were selected because of their significant experience and ability to recruit for PCI in cardiology as well as their ability to collaborate across disciplines to ensure that patients receive comprehensive treatment and rehabilitative support. The study protocol was reviewed and approved by the Ethics Committee of Qilu Hospital of Shandong University and other participating institutions (Ethics Committee reference number KYLL-202308-006). The study was registered on ClinicalTrials.gov, ID: NCT06640400.

Participants in this study will be recruited from the aforementioned medical institutions. Inclusion criteria call for hospitalized patients diagnosed (through laboratory and imaging tests) with CAD requiring PCI ^[16]^. Following consent from the patients or their family members, screening procedures will be conducted by medical professionals to ensure compliance with the eligibility criteria. Only those individuals meeting all recruitment criteria and no exclusion criteria will be included in the study.

**3.2 Participants: Inclusion and exclusion criteria**

**3.2.1 Inclusion Criteria:**

• Age at enrollment: 18 years or older;

• Confirmed CAD requiring elective PCI;

• Thrombolysis in myocardial infarction (TIMI) flow grade 2 or above after PCI;

• No intraoperative complications following PCI, such as entrapment, reflux, or perforation of the coronary artery;

• Intact skin in the anterior chest area;

• Cardiac enzymes and troponin I within normal range;

**3.2.2 Exclusion Criteria:**

• ST-segment elevation myocardial infarction and non–ST-segment elevation myocardial infarction;

• Occlusion of branch vessels during PCI;

• Perioperative use of hormones or immunosuppressants;

• Combined infection or other inflammatory diseases;

• Postoperative fever;

• Allergy to contrast media or cardiovascular ultrasound acoustic head–related materials;

• Changes in lipid-lowering, antiplatelet, and antihypertensive drug regimens during treatment;

• A clear diagnosis of autoimmune disease;

• A history of other surgeries or trauma in the preceding 7 days;

• Infection with the novel coronavirus during the preceding month;

• Previous PCI treatment;

• Previous cardiovascular ultrasound treatment;

• Diabetes mellitus.

**3.3 Criteria for the withdrawal of subjects from the trial**

**3.3.1 Subjects withdraw from the test on their own**

Patients can voluntarily withdraw from the trial at any time without providing any reason; or the subject has not explicitly withdrawn from the trial, but is no longer receiving LIPUS treatment or related indicators, it is also considered "withdrawal". Whenever possible, understand the reasons for the subject's withdrawal and document them in the case report form. Every effort should be made to keep the patient in the study and to continue with the required study-related treatments and markers.

For patients who voluntarily withdraw from the trial, the reason for withdrawal, the course of treatment, the time of the last treatment, and the results of the most recent test should be clearly recorded; The relationship between the branch or shedding in the trial and the trial should be carefully recorded, the possible impact of the discontinued case on the research conclusion should be analyzed, and the case report form should be completed and completed; Keep the original data of all exfoliated cases.

**3.3.2 Withdrawal/drop-off as determined by the investigator**

The withdrawal decided by the investigator refers to the fact that the enrolled subjects are not suitable to continue the trial during the trial, and the investigator decides to withdraw from the trial.

1) During the trial, there are changes in the subject's condition, such as acute stent thrombosis, malignant arrhythmia, contrast nephropathy, acute left heart failure, hemodynamic instability, etc., which affect the compliance of continuing to participate in the trial or continue to participate in the trial may affect the safety of the subject, and the investigator believes that the patient should withdraw;

2) Patients with a definite diagnosis of PCI-related myocardial infarction at 48h;

3) Subjects who violate the inclusion/exclusion criteria, or have poor protocol compliance.

**3.3.3 Early termination/closure of the test site**

Trial suspension refers to the fact that the clinical trial has not been completed as planned, and is stopped in the middle of the trial. Reasons for discontinuation of a clinical trial include, but are not limited to:

1) Serious safety issues occurred during the test;

2) In the trial, it was found that there were major errors in the clinical trial protocol, and although the protocol design was good, serious deviations occurred in the implementation;

3) The sponsor requests to suspend (such as financial reasons, management reasons, etc.);

4) Administrative reasons.

In the event of trial abortion, the safety and rights of the participants should be protected as much as possible. The investigator or other staff member cooperates as much as possible to complete the following tasks:

1) Submit all research data that have been generated;

2) Solve all data queries;

3) counting, checking and arranging all unused investigational products;

4) Review the completeness of the study records.

**3.4 Calculation of sample size**

Our calculations of sample size and power are based on the primary outcomes, utilizing a two-sided analysis with a statistical power of 90 % (1 – β = 0.90) and a significance level of α = 0.05. Owing to the lack of prior studies examining cardiovascular ultrasound therapy following PCI, the sample size was determined following an extensive evaluation by a panel of experts. Targeting long-term inflammation and cardiovascular efficacy, they selected high-sensitivity C-reactive protein (hsCRP) as the primary biomarker, given its relevance and the larger sample size required by interleukin 6.

Cardiovascular ultrasound is estimated to reduce hsCRP levels by an average of 4 mg/L. To meet this target, 160 participants (80 per group in a 1:1 allocation) are projected to be needed. Factoring in an anticipated dropout rate of 20 %, the final sample size would be adjusted to 200 participants (100 per group), ensuring adequate statistical power and robustness for the study’s objectives.

**3.5 Randomization and blinding**

Using a randomized 1:1 ratio, participants will be allocated into either of two groups: the cardiovascular ultrasound therapy group and the control group. This process will be facilitated by a computer-generated randomization sequence. To uphold the integrity of the allocation process, a team of research assistants not involved in clinical care or outcome assessment will meticulously craft sequentially numbered, opaque, sealed envelopes based on the predefined randomization list. This meticulous approach will ensure the safeguarding of confidentiality and the autonomy of allocation data. When appropriate, the assistants will open envelopes and ensure the coordination of therapeutic interventions.

Given the distinctive visual and operational characteristics of cardiovascular ultrasound therapy, the assignment results will not be concealed from the attending physicians or the patients. Only the investigative team members responsible for the collection of primary outcome data will remain masked. Consequently, the study design adheres to the framework of a nonblinded controlled trial. Patients will retain the prerogative to decline study participation, in which case they would receive standard care only.

**3.6 Informed consent**

The research assistants (CHs) will take informed consent before trial baseline evaluation in the participants’ reception room. Participants will be told why the study is being conducted, what they will be doing, and the possible benefits and risks. If participants have any questions, they will be free to ask them. Then, being fully informed, they will be able to decide whether or not to participate in the study.

**3.7 Interventions**

**3.7.1 Explanation for the choice of comparators**

We intend to select the prevailing standard (nonstimulating) allopathic therapy as the control condition to determine if the expected benefits of cardiovascular ultrasound therapy will produce better anti-inflammatory results than standard therapy.

**3.7.2 Intervention description**

Participants allocated to the intervention group will undergo a 10-day regimen utilizing a medical cardiovascular ultrasound therapy device (838C-M-L-I/II, Shenzhen, China). The ultrasound instrument is equipped with a sound head comprising five transducer units operating at an ultrasonic frequency of 0.84 MHz with a sound intensity range of 1 W/cm^2^–1.25 W/cm^2^. The therapeutic ultrasound sessions require a controlled environmental temperature. Patients will assume the supine position, exposing the precordial region, with the 5-pronged head positioned parallel to the heart’s long axis; it should cover the entire precordial region including the right and left coronary arterial trunks and the aortic root (Fig.1). The five transducer units function sequentially in a scanning pattern, with each unit operating for 5 seconds and transitioning with a 0.5-second interval, following a clockwise rotation until the treatment has been completed. The cardiovascular ultrasound therapy device operates in a pulsed mode, and each treatment session lasts for 20 minutes; there would be two daily sessions for a total of 20 treatments. The control group was not given extracorporeal cardiac ultrasound therapy. Participants in the control group will not receive cardiovascular ultrasound therapy. cardiovascular ultrasound therapy will be assessed at baseline (24th-hour post-PCI), on the 10th day, and at the end of the first and third months post-intervention. If it is necessary to change the medication regimen as much as possible, if it is necessary to change the medication regimen, it should be decided after evaluation by the investigator, and if the medication regimen that does need to be changed has an impact on the test results, the patient should be excluded and the reasons for changing the medication regimen should be recorded in detail.


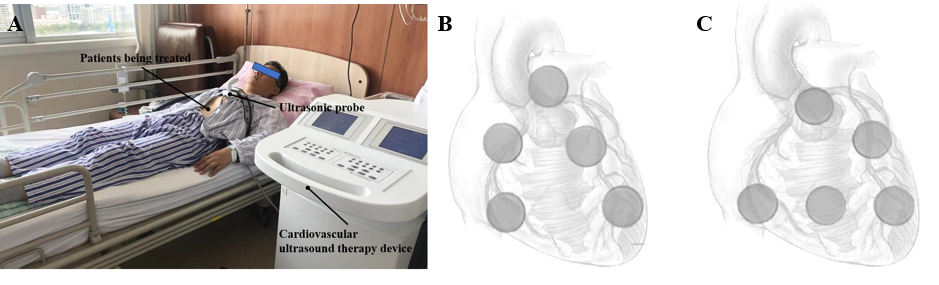


Fig. 1. Diagram of ultrasound head placement.

A: Patients undergoing cardiovascular ultrasound therapy. B is used in patients with occlusion of one coronary artery and C is used in patients with occlusion of both coronary arteries.

**3.7.3 Strategies to improve adherence to interventions**

To promote adherence to the interventions, free measures (including vascular endothelial function testing, hemodynamic monitoring, the 6-minute walk test [6MWT], HRV testing, recording of symptoms, and scoring of depression and anxiety) will be offered following treatment.

**3.8 Guarantee of treatment adherence**

Since this study was completed during the patient's hospitalization, the patient's treatment compliance could be educated by the supervising physician about the patient's compliance.

**3.9 Outcomes**

**3.9.1 Primary outcome**

The primary outcome measures will focus on the levels of hsCRP and interleukin-6 in the peripheral serum following 20 cardiovascular ultrasound therapy sessions (i.e., on the 10th day). Comparative analyses will be conducted between the cardiovascular ultrasound therapy intervention and control groups along with pre- and postintervention assessments.

**3.9.2 Secondary outcome**

**(1) Indicators of serum myocardial injury and blood lipid levels**

Serum biomarkers indicative of myocardial injury encompass creatine kinase isoenzymes (CK-MB), cardiac troponin I (c-TnI), and myeloperoxidase (MPO). Lipid profile markers in serum comprise total cholesterol (TC), triglycerides (TG), low-density lipoprotein cholesterol (LDL-c), high-density lipoprotein cholesterol (HDL-c), apolipoprotein A (ApoA), apolipoprotein B (ApoB), lipoprotein (a), and oxidized low-density lipoprotein (ox-LDL) in the peripheral blood.

Monitoring of serum biomarkers indicative of myocardial injury will be conducted at baseline (24th hour post PCI), on the 10th day, and at the end of the 1st and 3rd months post intervention.

**(2) Serum markers of endothelial function**

The serum markers utilized to assess endothelial function encompass endothelial nitric oxide synthase (eNOS), endothelin-1 (ET-1), and vascular endothelial growth factor (VEGF) in the peripheral blood.

Monitoring of serum endothelial function will be conducted at baseline (24th hour post PCI); on the 5th and 10th days; and at the end of the 1st and 3rd months post intervention.

**(3) Serum inflammatory factors**

Each morning, 5 mL of fasting venous blood will be drawn by nursing staff to assess the proportion of inflammatory cells and measure levels of inflammatory markers in the peripheral blood.

Monitoring of serum inflammatory factors will be conducted at baseline (24th hour post PCI), on the 10th day, and at the end of the 1st and 3rd months post intervention.

**(4) Hemodynamic parameters**

These encompass a comprehensive range of indicators, including cardiac index (CI), cardiac output (CO), stroke volume (SV), stroke volume index (SVI), mean arterial pressure (MAP), stroke systemic vascular resistance index (SSVRI), left ventricular stroke work index (LVSWI), systemic vascular resistance index (SVRI), ejection phase contraction index (EPCI), inotropic state index (ISI), systemic vascular resistance (SVR), vascular resistance (VR), and mean heart rate (mHR).

Certified sonographers will monitor hemodynamic parameters at baseline (24th hour post PCI); on the 5th and 10th days; and at the end of the 1st and 3rd months post intervention.

**(5) Echocardiography**

The echocardiogram parameters will encompass E/e' ratio, left ventricular ejection fraction (LVEF), and wall thickening fraction (WTF). WTF is defined as [(end-diastolic wall thickness − end-systolic wall thickness)/ end-diastolic wall thickness] × 100 (%).

Echocardiographic assessments will be conducted by certified sonographers proficient in thoracic echocardiography. This will be done at multiple time points: at baseline (24th hour post PCI), on the 5th and 10th days, and at the end of the 1st and 3rd months post intervention.

**(6) Ultrasound examination of carotid plaques**

Ultrasonography of carotid plaques includes their location, size (length × width), and morphology as well as the intima-media thickness (IMT) and Crouse’s score of the common carotid artery.

A certified sonographer proficient in examining the carotid arteries will perform ultrasound evaluations at multiple time points: at baseline (24th hour post PCI); on the 5th and 10th days; and at the end of the 1st and 3rd months post intervention.

**(7) Six-minute walk test**

The six-minute walk test (6MWT) quantifies the maximal distance an individual can walk in 6 minutes, serving as an indicator of the patient's exercise capacity and cardiorespiratory fitness during routine physical activities.

A certified cardiologist will conduct the 6MWT at various time points, including at enrollment, on the10th day, and at the end of the 1st and 3rd months post intervention.

**(8) Short-term heart rate variability**

HRV refers to the fluctuation in the timing of consecutive heartbeats or the variance in heart rate. It is determined by the duration between two successive R-R intervals, representing the slight deviation between each cardiac cycle. HRV assessment will be conducted using the amor HRV Guard Analyzer (ZSY-1, Taiwan, China). With the patient at rest and in a supine position, short-term (5-minute) HRV will be measured during daytime hours.

Certified cardiologists will perform short-term HRV assessments at baseline (24th hour post PCI), on the 5th and 10th days, and at the end of the 1st and 3rd months post intervention.

**(9) Tools for mental health assessment**

The Patient Health Questionnaire-9 (PHQ-9) is a critical instrument for the screening, diagnosis, and assessment of depression, enabling the measurement of its severity. Concurrently, the 7-item Generalized Anxiety Disorder Scale (GAD-7) enables the evaluation of generalized anxiety disorder. Similarly, the Pittsburgh Sleep Quality Index (PSQI) stands as a widely recognized assessment tool for determining sleep quality, and the Seattle Angina Questionnaire (SAQ) functions as a self-administered assessment tool designed to evaluate specific functional status and quality of life among individuals with CAD.

Mental health assessments will be conducted by certified psychologists at various time points: at baseline (24th hour post PCI), on the 10th day, and at the end of the 1st and 3rd months post intervention.

**4** **Statistical analysis**

The baseline characteristics of both groups will be summarized using appropriate descriptive statistics. Analysis of both primary and secondary outcomes will be based on the intention-to-treat approach, ensuring that all participants remain assigned to their original group throughout the study.

Normally distributed data will be presented as the mean (standard deviation), and non–normally distributed data will be depicted as the median (interquartile range). Group comparisons will be conducted using independent sample tests, and within-group comparisons before and after treatment will be evaluated using paired sample tests. Clinical symptom variables will be portrayed as percentages. Categorical data will be assessed using either the chi-squared test or Fisher’s exact test. A significance level of.05 will be employed to interpret the P values. The statistical analysis will be conducted utilizing SPSS version 26 statistical software (IBM, Armonk, NY).

**4.1 Interim analyses**

No interim analyses are being planned.

**4.2 Methods for additional analyses (e.g., subgroup analyses)**

No subgroup analyses will be conducted.

**4.3 Analytic methods for handling protocol nonadherence and statistical methods for handling missing data**

We anticipate minimal missing data (<10%) for the primary outcome given that all participants will be in patients with regular daily contact maintained during treatment. The evaluation of missing data effects will involve comparing differences under plausible and extreme missing data scenarios. In the event of a significant primary test result, we will also assess the interaction size between group assignment and missing data necessary to render the difference nonsignificant. Should 10% or more of the baseline samples be excluded due to missing data, we will apply multiple imputation techniques for comprehensive analysis.

**5 Technology roadmap**

The study protocol’s flowchart is outlined in Fig. 2.


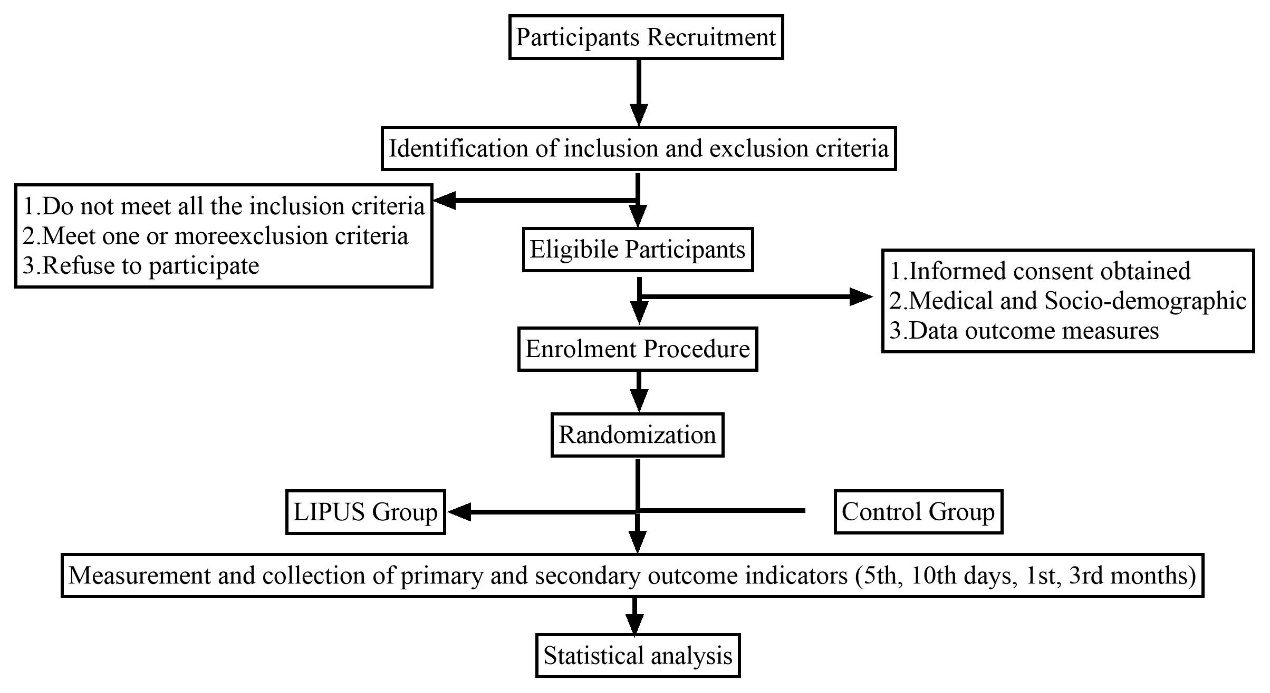


Fig. 2. Participant flowchart for cardiovascular ultrasound therapy

**6 Adverse events**

**6.1 Adverse events reporting and harms**

All adverse events, defined as any unfavorable or unintended reactions to the intervention, will be meticulously documented based on patient-reported symptoms and observational assessments during each visit. To date, no adverse events have been reported with use of the cardiovascular ultrasound therapy regimen in cardiovascular disease. Potential mild adverse reactions—including mild local swelling, heightened local pain response, and altered local sensitivity—will be monitored closely. In the event of such symptoms, prompt and suitable symptomatic treatment will be administered.

**6.2 Grading of the degree of adverse events**

The investigator assesses the severity of the AE according to CTCAEV5.0:

Grade 1: mild, asymptomatic, or mild; Seen clinically or diagnostically only; No treatment is required;

Grade 2: moderate, requiring smaller, topical or non-immersion treatment, age-appropriate instrumental daily activities (ADLs) limitation (instrumental ADLs refer to cooking, buying clothes, using the phone, managing money, etc.);

Grade 3: Severe or medically significant but not immediately life-threatening; resulting in hospitalization or prolongation of hospitalization; Disability; Limitation of self-rational activities of daily living (self-responsible activities of daily living refer to bathing, dressing and undressing, eating, washing, taking medication, etc., and not being bedridden;

Grade 4: life-threatening, requiring urgent treatment;

Grade 5: AE-related death.

**6.3 Methods for recording and reporting adverse events**

(1) The investigator should explain to the patient that it is necessary to truthfully reflect the changes in the condition after taking LIPUS and avoid inducing questions.

(2) While observing the efficacy, pay close attention to the observation of adverse reactions or unexpected toxic side effects (including symptoms, signs, laboratory examinations), regardless of whether they are related to LIPUS or not, should be recorded in detail, and the causes should be analyzed, judgments should be made, and follow-up observation and records should be followed. The incidence of adverse reactions should be counted.

(3) For adverse reactions occurring during the study, the symptoms, severity, occurrence time, duration, treatment measures, and process should be recorded in the study medical record, and their relevance to LIPUS treatment should be evaluated, and recorded in detail by the investigator, signed and dated.

(4) When adverse reactions are found, the observer physician can decide whether to suspend the observation according to the condition, and the cases that withdraw from the trial due to adverse reactions should be followed up and investigated, and the treatment process and results should be recorded in detail.

(5) If there is a serious adverse event in the study, the unit undertaking the clinical study must take immediate measures to protect the safety of the subjects and report to the sponsor within 24 hours or no later than the 2nd working day. The Sponsor will ensure that all reporting procedures required by laws and regulations are met.

**7 Oversight and monitoring**

**7.1 Composition of the coordinating center and trial steering committee**

Ongoing clinical monitoring will be overseen by a day-to-day management team comprising the principal investigator (PI), a doctoral student, a research coordinator, and an assistant manager. Regular weekly meetings will be held by the group to ensure effective coordination and management of the study. This trial does not have a trial steering committee.

**7.2 Composition of the data monitoring committee—its role and reporting structure**

No formal data monitoring committee has been established for this trial, as there are no interim analyses or planned procedures for early stopping.

**7.3 Frequency and plans for auditing trial conduct**

Not applicable. No auditing is planned in this trial. The PI will meet weekly during the study period to review trial progress.

**7.4 Plans for communicating important protocol amendments to relevant parties (e.g., trial participants, ethical committees)**

Any proposed modifications to the study protocol require prior approval from the Ethics Committee of Qilu Hospital of Shandong University. Upon approval, these protocol amendments must be documented in the trial register and, for transparency and accuracy, integrated into the final research report.

**7.5 Dissemination plans**

The outcomes of the clinical trial are intended for dissemination through publication in medical journals and presentations at national and international conferences. The lead and corresponding authors will be responsible for overseeing this process.

**8 References**

[1] Stone PH, Libby P, Boden WE. Fundamental Pathobiology of Coronary Atherosclerosis and Clinical Implications for Chronic Ischemic Heart Disease Management-The Plaque Hypothesis: A Narrative Review. JAMA Cardiol. 2023;8(2):192-201. https://doi.org/10.1001/jamacardio.2022.3926 PMID: 36515941.

[2] Roth GA, Mensah GA, Johnson CO, Addolorato G, Ammirati E, Baddour LM, et al. Global Burden of Cardiovascular Diseases and Risk Factors, 1990-2019: Update From the GBD 2019 Study. J Am Coll Cardiol. 2020;76(25):2982-3021. https://doi.org/10.1016/j.jacc.2020.11.010 PMID: 33309175.

[3] Hoole SP, Bambrough P. Recent advances in percutaneous coronary intervention. Heart. 2020;106(18):1380-1386. doi: 10.1136/heartjnl-2019-315707. https://doi.org/10.12968/hosp.2003.64.3.1793 PMID: 32522821.

[4] Ozaki Y, Tobe A, Onuma Y, Kobayashi Y, Amano T, Muramatsu T, et al. CVIT expert consensus document on primary percutaneous coronary intervention (PCI) for acute coronary syndromes (ACS) in 2024. Cardiovasc Interv Ther. 2024;39(4):335-375. https://doi.org/10.1007/s12928-024-01036-y PMID: 39302533.

[5] Tao S, Tang X, Yu L, Li L, Zhang G, Zhang L, et al. Prognosis of coronary heart disease after percutaneous coronary intervention: a bibliometric analysis over the period 2004-2022. Eur J Med Res. 2023;28(1):311. https://doi.org/10.1186/s40001-023-01220-5 PMID: 37658418.

[6] Cruz Rodriguez JB, Kar S. Management of Angina Post Percutaneous Coronary Intervention. Curr Cardiol Rep. 2020;22(2):7. https://doi.org/10.1007/s11886-020-1259-9 PMID: 31965355.

[7] Jiang X, Savchenko O, Li Y, Qi S, Yang T, Zhang W, et al. A Review of Low-Intensity Pulsed Ultrasound for Therapeutic Applications. IEEE Trans Biomed Eng. 2019;66(10):2704-2718. https://doi.org/10.1109/TBME.2018.2889669 PMID: 30596564.

[8] Xin Z, Lin G, Lei H, Lue TF, Guo Y. Clinical applications of low-intensity pulsed ultrasound and its potential role in urology. Transl Androl Urol. 2016;5(2):25566. https://doi.org/10.21037/tau.2016.02.04 PMID: 27141455.

[9] Hanawa K, Ito K, Aizawa K, Shindo T, Nishimiya K, Hasebe Y, et al. Low-intensity pulsed ultrasound induces angiogenesis and ameliorates left ventricular dysfunction in a porcine model of chronic myocardial ischemia. PLoS One. 2014;9(8):e104863. https://doi.org/10.1371/journal.pone.0104863 PMID: 25111309.

[10] Cao Q, Liu L, Hu Y, Cao S, Tan T, Huang X, et al. Low-intensity pulsed ultrasound of different intensities differently affects myocardial ischemia/reperfusion injury by modulating cardiac oxidative stress and inflammatory reaction. Front Immunol. 2023; 14:1248056. https://doi.org/10.3389/fimmu.2023.1248056 PMID: 37744362.

[11] Weng L, Li L, Zhao K, Xu T, Mao Y, Shu H, et al. Non-Invasive Local Acoustic Therapy Ameliorates Diabetic Heart Fibrosis by Suppressing ACE-Mediated Oxidative Stress and Inflammation in Cardiac Fibroblasts. Cardiovasc Drugs Ther. 2022;36(3):413-424.https://doi.org/10.1007/s10557-021-07297-6 PMID: 35156147.

[12] Zhang B, Chen H, Ouyang J, Xie Y, Chen L, Tan Q, et al. SQSTM1-dependent autophagic degradation of PKM2 inhibits the production of mature IL1B/IL-1β and contributes to LIPUS-mediated anti-inflammatory effect. Autophagy. 2020 Jul;16(7):1262-1278. https://doi.org/10.1080/15548627.2019.1664705 PMID: 31500508.

[13] He YF, Wang XL, Deng SP, Wang YL, Huang QQ, Lin S, et al. Latest progress in low-intensity pulsed ultrasound for studying exosomes derived from stem/progenitor cells. Front Endocrinol (Lausanne). 2023;14:1286900. https://doi.org/10.3389/fendo.2023.1286900 PMID: 38089611.

[14] Shindo T, Ito K, Ogata T, Kurosawa R, Eguchi K, Kagaya Y, et al. A randomized, double-blind, placebo-controlled pilot trial of low-intensity pulsed ultrasound therapy for refractory angina pectoris. PLoS One. 2023;18(6):e0287714. https://doi.org/10.1371/journal.pone.0287714 PMID: 37352324.

[15] Mohamad Yusoff F, Kajikawa M, Yamaji T, Kishimoto S, Maruhashi T, Nakashima A, et al. Low-intensity pulsed ultrasound improves symptoms in patients with Buerger disease: a double-blinded, randomized, and placebo-controlled study. Sci Rep. 2024;14(1):13704. https://doi.org/10.1038/s41598-024-64118-0 PMID: 38871832.

[16] Fihn SD, Gardin JM, Abrams J, Berra K, Blankenship JC, Dallas AP, et al. 2012 ACCF/AHA/ACP/AATS/PCNA/SCAI/STS guideline for the diagnosis and management of patients with stable ischemic heart disease: a report of the American College of Cardiology Foundation/American Heart Association task force on practice guidelines, and the American College of Physicians, American Association for Thoracic Surgery, Preventive Cardiovascular Nurses Association, Society for Cardiovascular Angiography and Interventions, and Society of Thoracic Surgeons. Circulation. 2012;126(25):e354-471. https://doi.org/10.1161/CIR.0b013e318277d6a0 PMID: 23166211.
