# Supplementary material for: A study protocol of the rehabilitative efficacy of cardiovascular ultrasound therapy after percutaneous coronary intervention in patients with coronary artery disease: A multicenter, parallel-group, randomized controlled study
Source: PLoS One. 2025 Oct 16;20(10):e0327557. doi: 10.1371/journal.pone.0327557 (PMC12530608; doi:10.1371/journal.pone.0327557)
Supplement: S3 File — (DOCX) [file pone.0327557.s003.docx]

**Research Ethics Committee of Qilu Hospital of Shandong University**

**Ethical Review Approval**

| Ethical Approval Number | KYLL-202308-006 | | |
| --- | --- | --- | --- |
| Project name | A study of the rehabilitative efficacy of cardiovascular ultrasound therapy after percutaneous coronary intervention in patients with coronary artery disease | | |
| Project source | Horizontal Project | | |
| Research section | Department of Geriatric Medicine | | |
| Project Manager | Lin Shen | | |
| Type of review | Follow-up review | Modalities of the review | Expedited review |
| Date of review | September 13, 2024 | | |
| Review of documentation | Progress reports on research | | |
| Review comments:  In accordance with the ethical principles of China's Measures for Ethical Review of Biomedical Research Involving Human Beings, the WMA Declaration of Helsinki and the CIOMS International Ethical Guidelines for Biomedical Research on Human Beings, this Ethics Committee has reviewed and agreed to continue this study.  1. Please follow the protocol approved by the Ethics Committee to conduct the clinical research and protect the health and rights of the subjects.  2. Before starting the study, please complete the clinical research record.  3. If there is any change of principal investigator during the study and any modification to the clinical study protocol, informed questionnaire, recruitment materials, etc., the applicant is requested to submit an application for amendment review.  4. When a serious adverse event occurs, the applicant is requested to submit a serious adverse event report in a timely manner.  5. In accordance with the frequency of annual/periodic follow-up checks set by the Ethics Committee, the applicant is requested to submit a report on the progress of the study one month before the deadline; the sponsor should submit a summary report on the progress of the study in each center to the Ethics Committee of the head unit of the group; the applicant is requested to submit a written report to the Ethics Committee in a timely manner when there is any circumstance that may significantly affect the conduct of the study or increase the risk to the subjects.  6. The sponsor/monitor/investigator is requested to submit a protocol violation report if the study includes subjects who do not meet the inclusion criteria or meet the exclusion criteria, fails to enter or exit the study despite meeting the requirements for discontinuation of the study, administers incorrect treatments or dosages, or administers combinations prohibited by the sub-protocol, or if the ethical principles are violated in a way that may adversely affect the rights/health of the subjects and the scientific validity of the study. The investigator to submit a report on the violation of the protocol.  7. If the applicant suspends or prematurely terminates the clinical study, the applicant is requested to submit a report on the suspension/termination of the study in a timely manner.  8. Upon completion of the clinical study, the applicant is requested to submit a completion report. | | | |
| Frequency of annual/periodic follow-up review | 12 months | | |
| Valid period | September 14, 2024 to September 14, 2025 | | |
| Signature of Chairperson | Lin Shen | | |
| Ethics Committee | Research Ethics Committee of Qilu Hospital of Shandong University | | |
| Date | September 13, 2024 | | |
