# Supplementary material for: A study protocol of the rehabilitative efficacy of cardiovascular ultrasound therapy after percutaneous coronary intervention in patients with coronary artery disease: A multicenter, parallel-group, randomized controlled study
Source: PLoS One. 2025 Oct 16;20(10):e0327557. doi: 10.1371/journal.pone.0327557 (PMC12530608; doi:10.1371/journal.pone.0327557)
Supplement: S1 Table — (DOCX) [file pone.0327557.s004.docx]

**S1 Table: The Patient Health Questionnaire (PHQ-9)**

Patient Name

Date of Visit

| **Over the past 2 weeks, how often have you been bothered by any of the following problems?** | **Not**  **at all** | **Several**  **days** | **More**  **than half**  **the days** | **Nearly**  **every**  **day** |
| --- | --- | --- | --- | --- |
| 1. Little interest or pleasure in doing things | 0 | 1 | 2 | 3 |
| 2. Feeling down, depressed or hopeless | 0 | 1 | 2 | 3 |
| 3. Trouble falling asleep, staying asleep, or sleeping too much | 0 | 1 | 2 | 3 |
| 4. Feeling tired or having little energy | 0 | 1 | 2 | 3 |
| 5. Poor appetite or overeating | 0 | 1 | 2 | 3 |
| 6. Feeling bad about yourself - or that you’re a failure or have let yourself or your family down | 0 | 1 | 2 | 3 |
| 7. Trouble concentrating on things, such as reading the newspaper or watching television | 0 | 1 | 2 | 3 |
| 8. Moving or speaking so slowly that other people could have noticed. Or, the opposite - being so fidgety or restless that you have been moving around a lot more than usual | 0 | 1 | 2 | 3 |
| 9. Thoughts that you would be better off dead or of hurting yourself in some way | 0 | 1 | 2 | 3 |

Column Totals + + Add Totals Together

10. If you checked off any problems, how difficult have those problems made it for you to Do your work, take care of things at home, or get along with other people?


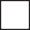
Not difficult at all
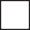
Somewhat difficult
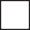
Very difficult


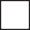
 Extremely difficult

Source: Kroenke K, Spitzer RL, Williams JB. The PHQ-9: validity of a brief depression severity measure. J Gen Intern Med. 2001;16(9):606-613. doi:10.1046/j.1525-1497.2001.016009606.x
