# Supplementary material for: A study protocol of the rehabilitative efficacy of cardiovascular ultrasound therapy after percutaneous coronary intervention in patients with coronary artery disease: A multicenter, parallel-group, randomized controlled study
Source: PLoS One. 2025 Oct 16;20(10):e0327557. doi: 10.1371/journal.pone.0327557 (PMC12530608; doi:10.1371/journal.pone.0327557)
Supplement: S2 Table — (DOCX) [file pone.0327557.s005.docx]

**S2 Table: Generalized Anxiety Disorder 7-item (GAD-7) scale**

| Over the last 2 weeks, how often have you been bothered by the following problems? | Not at | Several | Over half | Nearly |
| --- | --- | --- | --- | --- |
|  | all sure | days | the days | every day |
| 1. Feeling nervous, anxious, or on edge | 0 | 1 | 2 | 3 |
| 1. Not being able to stop or control worrying | 0 | 1 | 2 | 3 |
| 1. Worrying too much about different things | 0 | 1 | 2 | 3 |
| 1. Trouble relaxing | 0 | 1 | 2 | 3 |
| 1. Being so restless that it's hard to sit still | 0 | 1 | 2 | 3 |
| 1. Becoming easily annoyed or irritable | 0 | 1 | 2 | 3 |
| 1. Feeling afraid as if something awful might happen | 0 | 1 | 2 | 3 |
| Add the score for each column |  |  |  |  |
| Total Score (add your column scores) |  | | | |

If you checked off any problems, how difficult have these made it for you to do your work, take care of things at home, or get along with other people?

Not difficult at all

Somewhat difficult

Very difficult

Extremely difficult

**Scoring**

Scores of 5, 10, and 15 are taken as the cut-off points for mild, moderate and severe anxiety, respectively. When used as a screening tool, further evaluation is recommended when the score is 10 or greater.

Using the threshold score of 10, the GAD-7 has a sensitivity of 89% and a specificity of 82% for GAD. It is moderately good at screening three other common anxiety disorders - panic disorder (sensitivity 74%, specificity 81%), social anxiety disorder (sensitivity 72%, specificity 80%) and post-traumatic stress disorder (sensitivity 66%, specificity 81%).

Source: Spitzer RL, Kroenke K, Williams JBW, Lowe B. A brief measure for assessing generalized anxiety disorder. Arch Inern Med. 2006:166:1092-1097.
