# Supplementary material for: A study protocol of the rehabilitative efficacy of cardiovascular ultrasound therapy after percutaneous coronary intervention in patients with coronary artery disease: A multicenter, parallel-group, randomized controlled study
Source: PLoS One. 2025 Oct 16;20(10):e0327557. doi: 10.1371/journal.pone.0327557 (PMC12530608; doi:10.1371/journal.pone.0327557)
Supplement: S4 Table — (DOCX) [file pone.0327557.s007.docx]

**S4 Table: The Seattle Angina Questionnaire-7**

| 1. The following is a list of activities that people often do during the week. Although for some people with several medical problems it is dificult to determine what it is that limits them, please go over the activities listed below and indicate how much limitation you have had due to chest pain, chest tightness or angina over the past 4 weeks | |
| --- | --- |
|  | Activity |
| a. Walking indoors on level ground | □Extremely limited  □Quite a bit limited  □Moderately Limited  □Slightly limited  □Not at alllimited  □Limited for other reasons or did not do the activity |
| b. Gardening, vacuuming or carrying groceries | □Extremely limited  □Quite a bit limited  □Moderately Limited  □Slightly limited  □Not at alllimited  □Limited for other reasons or did not do the activity |
| c. Lifting or moving heavy objects (e.g. furniture, children) | □Extremely limited  □Quite a bit limited  □Moderately Limited  □Slightly limited  □Not at alllimited  □Limited for other reasons or did not do the activity |
| 2. Over the past 4 weeks, on average, how many times have you had chest pain, chest tightness or angina? I have had chest pain, chest tightness or angina... | □4 or more times per day  □1-3 times per day  □3 or more times per week but not every day  □1-2 times per week  □Less than once a week  □None over the past 4 weeks |
| 3. Over the past 4 weeks, on average, how many times have you had to take nitroglycerin (nitroglycerin tablets or spray) for your chest pain, chest tightness or angina?  I have taken nitroglycerin… | □4 or more times per day  □1-3 times per day  □3 or more times per week but not every day  □1-2 times per week  □Less than once a week  □None over the past 4 weeks |
| 4. Over the past 4 weeks, how much has your chest pain, chest tightness or angina limited your enjoyment of life? | □It has extremely limited my enjoyment of life  □It has limited my enjoyment of life quite a bit  □It has moderately limited my enjoyment of life  □It has slightly limited my enjoyment of life  □It has not limited my enjoyment of life at all |
| 5. lf you had to spend the rest of your life with your chest pain, chest tightness or angina the way it is right now, how  would you feel about this? | □Not satisfied at all  □Mostly dissatisfied  □Somewhat satisfied  □Mostly satisfied  □Completely satisfied |

**S4 Table** The shortened SAQ-7 instrument. Although the original Seattle Angina Questionnaire (SAQ) instrument was designed to independently assess patients’ symptoms, function, and quality of life, the 19 items of the SAQ made interpretation more complex and less feasible to administer. In this figure, we present the shortened SAQ-7 instrument, with similar construct validity, predictiveness, reliability, and responsiveness as the original SAQ instrument.

Source: Chan PS, Jones PG, Arnold SA, Spertus JA. Development and validation of a short version of the Seattle angina questionnaire. Circ Cardiovasc Qual Outcomes. 2014;7(5):640-647.
